# Evaluating the Antibacterial and Antivirulence Activities of Floxuridine against *Streptococcus suis*

**DOI:** 10.3390/ijms241814211

**Published:** 2023-09-18

**Authors:** Jie Li, Ning Han, Yangyang Li, Feifei Zhao, Wenguang Xiong, Zhenling Zeng

**Affiliations:** 1 Guangdong Provincial Key Laboratory of Veterinary Pharmaceutics Development and Safety Evaluation, College of Veterinary Medicine, South China Agricultural University, Guangzhou 510642, China; xjzxlj@126.com (J.L.); xiongwg@scau.edu.cn (W.X.); 2 National Laboratory of Safety Evaluation (Environmental Assessment) of Veterinary Drugs, College of Veterinary Medicine, South China Agricultural University, Guangzhou 510642, China; 3 National Risk Assessment Laboratory for Antimicrobial Resistance of Animal Original Bacteria, College of Veterinary Medicine, South China Agricultural University, Guangzhou 510642, China

**Keywords:** floxuridine, *Streptococcus suis*, mode of action, anti-virulence

## Abstract

*Streptococcus suis* is an emerging zoonotic pathogen that can cause fatal diseases such as meningitis and sepsis in pigs and human beings. The overuse of antibiotics is leading to an increased level of resistance in *S. suis*, and novel antimicrobial agents or anti-virulence agents for the treatment of infections caused by *S. suis* are urgently needed. In the present study, we investigated the antibacterial activity, mode of action and anti-virulence effects of floxuridine against *S. suis*. Floxuridine showed excessive antibacterial activity against *S. suis* both in vivo and in vitro; 4 × MIC of floxuridine could kill *S. suis* within 8 h in a time–kill assay. Meanwhile, floxuridine disrupted the membrane structure and permeability of the cytoplasmic membrane. Molecular docking revealed that floxuridine and SLY can be directly bind to each other. Moreover, floxuridine effectively inhibited the hemolytic capacity and expression levels of the virulence-related genes of *S. suis*. Collectively, these results indicate that the FDA-approved anticancer drug floxuridine is a promising agent and a potential virulence inhibitor against *S. suis*.

## 1. Introduction

*Streptococcus suis* is a major pathogen in pigs, causing a wide range of diseases including arthritis, meningitis and sepsis [1,2]. Moreover, the transmission of *S. suis* to humans can occur through direct contact with infected pigs or carriers [3]. The first case of human infection with *S. suis* was reported in Denmark in 1968 [4]. Subsequently, the emergence of *S. suis* infections in most of Western Europe and several other East and Southeast Asian countries posed serious threats to public health [5]. Therefore, *S. suis* infections not only incur serious economic losses in the swine production sector but also pose a significant risk of human infection, particularly among individuals employed in the pig industrial chain [1,6]. Nevertheless, the overuse and misuse of antibiotics have led to a growing problem of bacterial drug resistance on a global scale [7]. *S. suis* is also considered to be an antimicrobial resistance reservoir, promoting the spread of antibiotic resistance genes to other streptococcal pathogens [8,9]. Currently, the antibiotic resistance of *S. suis* to tetracyclines [10,11], lincosamides [12,13] and macrolides [13,14] has spread worldwide. Despite the increasing number of infections caused by drug-resistant bacteria, the quantitative supply of new antibiotics is shrinking [15]. Facing a shortage of clinically effective antibiotics, there is an urgent demand for alternative strategies or novel antibiotics to effectively combat infections caused by *S. suis*.

Nowadays, the development of new drugs involves a great deal of time, effort, scientific research, and expense, mainly due to bottlenecks in the development process [16]. Drug repurposing has emerged as a promising and attractive approach to providing novel antimicrobials from FDA-approved drugs and compounds that are approved in other directions in the clinic [17,18,19]. Compared with the conventional discovery of new antibiotics, drug utilization reduces costs and eliminates the need for time-consuming clinical trials [20,21]. Nucleoside antibiotics are a large family of microbial secondary metabolites that are derived from simple cells [18]. They are used in the treatment of both cancer and viral infections because of their involvement in cellular processes such as DNA and RNA synthesis, enzyme regulation and metabolism [22]. Nucleoside antibiotics offer great chemical diversity, opening a new era that can be exploited for the development of antibacterial activity [18,19]. 

At present, many nucleotide analogues have been reported to have antibacterial activity against various bacteria [23]. Fluoropyrimidines are commonly used chemotherapy drugs for various forms of cancer, including kidney cancer and colorectal cancer [24,25]. Floxuridine demonstrates antitumor efficacy through its interference with DNA and RNA synthesis while also inducing thymineless cell death by inhibiting thymidylate synthase [26]. Floxuridine is a clinically used fluoropyrimidine drug that was approved by the FDA in 1970 [27]. The advantage of floxuridine is that it has a short half-life, a steep dose–response curve, a high total body clearance and a high level of hepatic extraction, making it a perfect drug for hepatic arterial infusion [24]. Some studies in recent years have reported the preliminary effects of floxuridine on bacteria [28,29]. However, the mechanism of antibacterial action is unclear. In this study, we demonstrate that floxuridine exerts a potential antibacterial activity against *S. suis* both in vitro and in vivo by disrupting the permeability of the cytoplasmic membrane (CM). In addition, floxuridine inhibits the virulence of *S. suis* by decreasing the expression of virulence-related genes.

## 2. Results

### 2.1. The Antibacterial Activity of Floxuridine 

**Figure 1:**
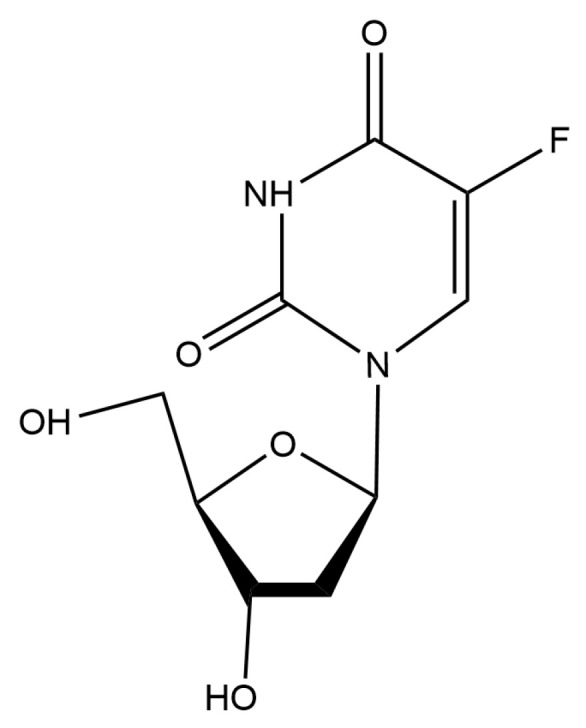
The chemical structure of floxuridine.

The antibacterial activity of floxuridine (Figure 1) against *S. suis* strains was detected via MIC in vitro. We used clinical isolates of *S. suis* with different multilocus sequence typing (MLST) types to determine the antibacterial activity of floxuridine. As shown in Table 1, floxuridine exhibited potential antibacterial activity against all *S. suis* strains regardless of MLST type, and the MIC values ranged from 0.06 to 0.5 μg/mL. These results suggested that floxuridine is a potential antimicrobial agent.

**Figure 2:**
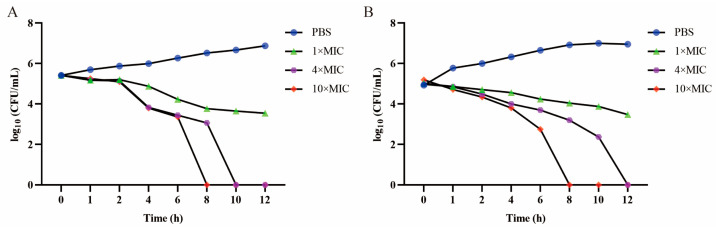
Time–kill assays of *S. suis* ATCC 43765 (**A**) and *S. suis* SS3 (**B**) after being co-cultured with different doses of floxuridine. The PBS is a negative control.

To further confirm the antibacterial effect against *S. suis*, a time–kill assay was conducted. We found that the antibacterial activity of floxuridine against *S. suis* was dose-dependent. After treatment with floxuridine at 10 × MIC, both *S. suis* ATCC 43765 and *S. suis* SS3 were completely eliminated within 8 h. After treatment with 4 × MIC of floxuridine, *S. suis* ATCC 43765 and *S. suis* SS3 were completely eliminated within 10 h and 12 h, respectively (Figure 2). These results indicated that floxuridine had a powerful antibacterial effect.

### 2.2. The Antihemolysin Activity of Floxuridine against S. suis

**Figure 3:**
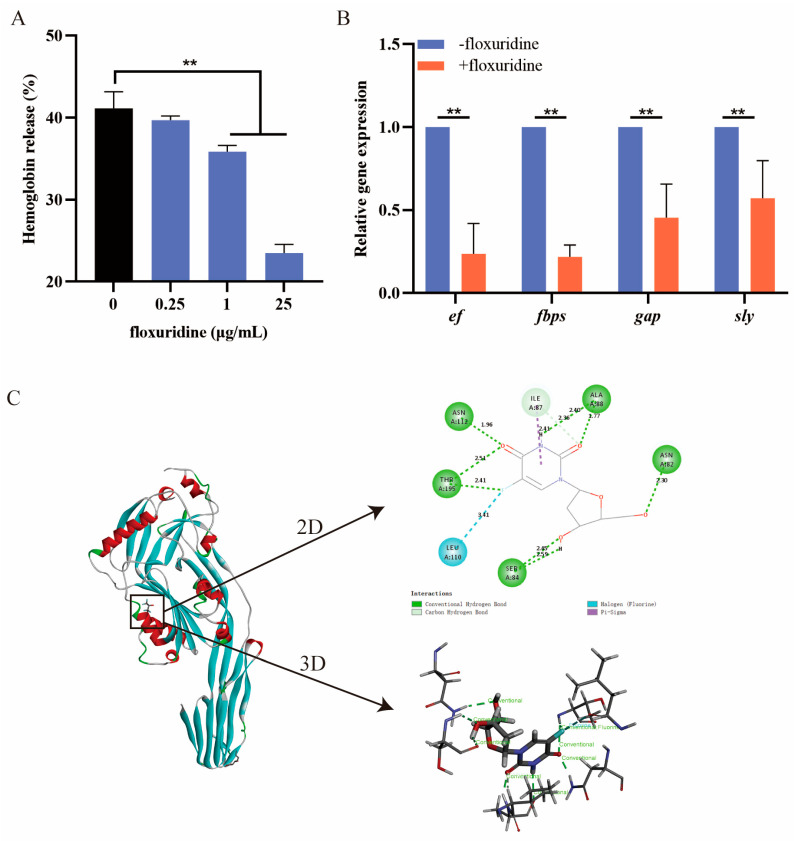
The inhibitory effect of floxuridine on the virulence of *S. suis*. (**A**) The hemolytic effect of floxuridine against an *S. suis* supernatant was determined at OD_543_ using sheep erythrocytes; 2.5% Triton X-100 was used as the positive control, and PBS was used as the negative control. (**B**) The expression levels of virulence-related genes (*ef*, *fabps*, *gapdh* and *sly*) of *S. suis* were significantly reduced after floxuridine treatment. All data are presented as means ± SD; ** *p* < 0.01. (**C**) The 2D and 3D structures with the binding sites of SLY complexed with floxuridine.

The effect of floxuridine on the hemolytic activity of *S. suis* was assessed. After treatment with different concentrations of floxuridine, the hemolytic ability of the *S. suis* supernatant, tested using sheep erythrocytes, was significantly reduced in a dose-dependent manner (Figure 3A), indicating that floxuridine can inhibit the hemolytic ability of *S. suis*. We then used a qRT-PCR to compare the expression levels of virulence-related genes after treatment with or without floxuridine. After treatment with 10 × MIC floxuridine, the expression levels of the genes *ef*, *gapdh*, *fbps* and *sly* were significantly reduced (Figure 3B). The binding site of floxuridine and SLY was further evaluated via molecular docking. The mode of action of the binding of floxuridine and SLY was demonstrated in 2D and 3D structures (Figure 3C). The potential ability to bind to the SLY protein was confirmed, with a binding energy of −3.88 kcal/mol. ASN112, THR195, LEU110, SER84, ILE87, ALA88 and ASN82 were identified as potential binding residues (Figure 3C). These results indicated that floxuridine may inhibit the virulence of *S. suis* by binding directly to the SLY gene and decreasing the expression of virulence-related genes.

### 2.3. Mode of Action of Floxuridine

**Figure 4:**
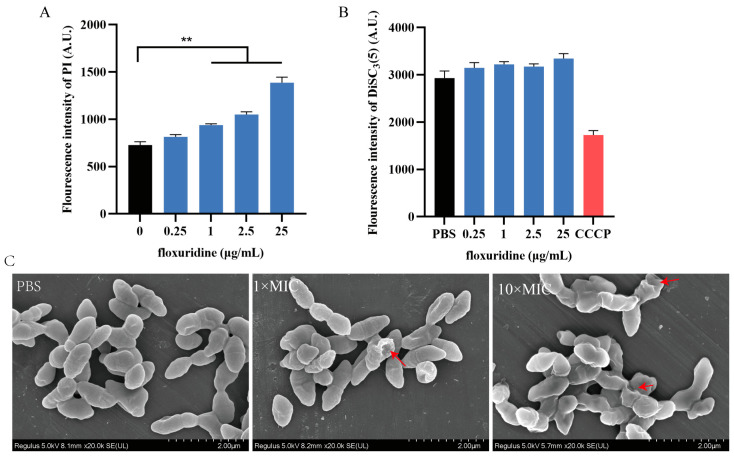
Mechanism of action of floxuridine against *S. suis*. (**A**) The permeability of the CM of *S. suis* was increased after treatment with floxuridine. Fluorescence was measured using a PI probe. (**B**) The membrane potential of *S. suis* was detected via fluorescence dye DiSC_3_(5). (**C**) SEM observation of the morphological changes in *S. suis* treated with different concentrations of floxuridine. Destroyed bacterial membrane was marked by red arrows. All data are presented as means ± SD; ** *p* < 0.01.

To further explore the antibacterial mechanism of floxuridine, we used a propidium iodide (PI) probe to measure the permeability of the bacterial CM. We found that the PI fluorescence increased in a dose-dependent manner with the floxuridine concentration (Figure 4A), indicating that floxuridine could disrupt the CM. In order to confirm the damage to the bacterial membrane, a scanning electron microscope was used to visualize the bacterial morphology after its treatment with floxuridine. We found that intact cell membrane structures were demonstrated by untreated bacteria; however, the shapes of the bacteria became irregular, raised or collapsed, and the bacterial surfaces appeared rough after their treatment with floxuridine (Figure 4C). Next, the bacterial membrane potential was evaluated using 3,3-dipropyl-thiadicarbocyanine (DiSC_3_(5)). Slight changes in fluorescence values were observed after the addition of floxuridine to DiSC_3_(5)-probed cells (Figure 4B), suggesting that the floxuridine disrupted the permeability of the CM but had no effect on the functionality of the membrane. 

### 2.4. In Vivo Efficacy

**Figure 5:**
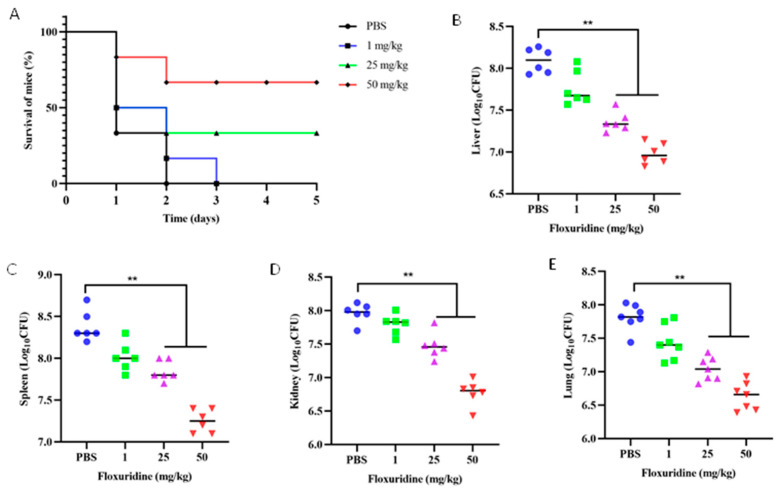
Evaluation of the therapeutic effect of floxuridine for the treatment of *S. suis* infections in vivo. (**A**) The floxuridine increased the survival rate of peritonitis–sepsis mice infected with *S. suis* in a dose-dependent manner. (**B**–**E**) Decreased bacterial loads in different organs (liver, spleen, kidney, and lung) of mice in the peritonitis–sepsis model. *p*-values were evaluated via a one-way ANOVA among multiple comparisons; ** *p* < 0.01.

**Figure 6:**
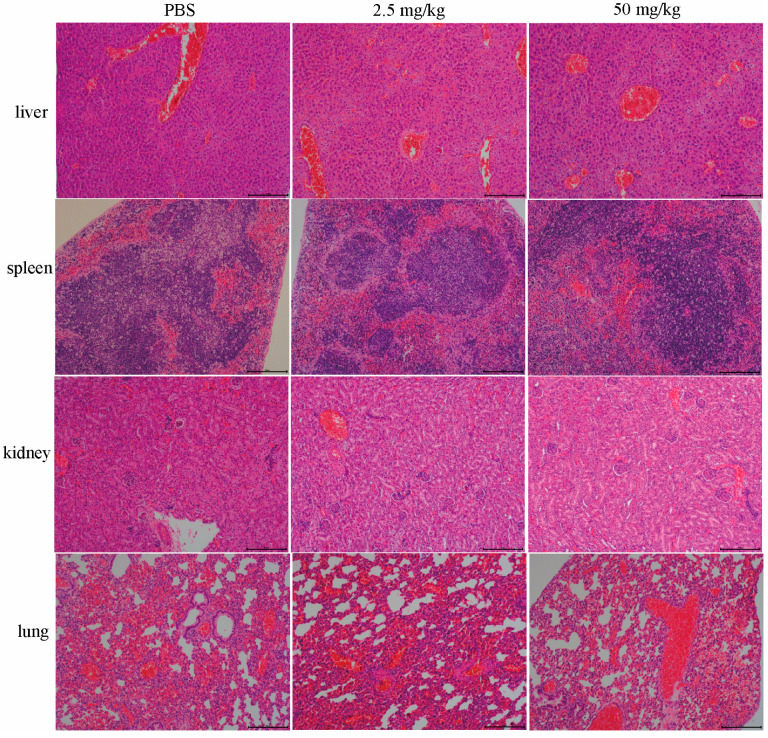
Histologic analysis of different organs (liver, spleen, kidney, and lung) of mice in the peritonitis–sepsis model. The livers, spleens, kidneys, and lungs of mice in different treatment groups were observed via hematoxylin–eosin (HE) staining. Following treatment with different concentrations of floxuridine, the pathological changes in the organs in the mice were alleviated. Scar bar, 200 μm.

Given the potent antibacterial activity of floxuridine against *S. suis* in vitro, we next evaluated its therapeutic efficacy in vivo using a murine peritonitis model. Mice were treated with floxuridine at 1 h post infection. We found that the mice without treatment all died within 2 d, while the mice treated with 25 and 50 mg/kg of floxuridine survived. Compared with the PBS group, the survival rate of the mice after the 50 mg/kg floxuridine treatment increased to 66.7% (Figure 5A). Moreover, the bacterial loads of different organs of mice in different treatment groups were detected. Compared with the untreated group, the bacterial loads in the lungs, kidneys, livers and spleens of the mice were also significantly reduced (Figure 5B–E). In the PBS group, the pulmonary tissue exhibited alterations characterized by the infiltration of inflammatory cells and congestion. The spleen displayed pronounced congestion and necrosis. A histopathological analysis of the liver revealed hepatocyte swelling and focal necrosis. Furthermore, the kidney exhibited renal tubular epithelial cell swelling and tubular necrosis. In contrast, the pathological changes in the organs of the mice in the treatment group were alleviated (Figure 6). These results showed that floxuridine was effective in the treatment of infections caused by *S. suis* in vivo.

## 3. Discussion

*S. suis* is a zoonotic pathogen that causes sepsis and meningitis in pigs and humans [30,31]. Streptococcus swine disease is not only a major cause of death and economic loss in the global pig industry but is also a threat to human health [7,32]. Nowadays, drug resistance in *S. suis* is a serious global problem, and new antibacterial strategies are urgently needed [33]. 

Drug repurposing is a potential strategy for the identification of new uses for old drugs. In recent years, a number of non-antibiotic drugs have been reported to have antibacterial effects. It is reported that robenidine, an anticoccidial drug used in poultry and rabbits, exhibited antibacterial activity against methicillin-resistant *Staphylococcus aureus* and vancomycin-resistant *Enterococcus* [34]. The antidiabetic drug metformin can effectively enhance the antibacterial activity of tetracycline antibiotics against MDR bacteria [35]. FDA-approved neurohormone melatonin is effective at improving the survival rate of mice and in reducing the bacterial load in the lungs during infection [36]. In this study, we found that the anticancer drug floxuridine exhibited potent antibacterial activity against *S. suis*. In fact, the antibacterial activity of floxuridine against *S. suis* was reported recently in a preliminary investigation of floxuridine as a promising antibacterial agent against *S. suis* which used the MIC and a *G. mellonella* larval model [28]. These results provide a preliminary rationale for the possible role of floxuridine in *S. suis* infection. In this study, we further investigated the potential antibacterial mechanism of floxuridine and its antihemolytic activity against *S. suis*. PI, a fluorescent probe that enhances fluorescence by combining the DNA in the cytoplasm, was used to measure the permeability of the CM [37]. After treatment with floxuridine, we found that floxuridine increased the permeability of the CM and disturbed the membrane morphology of *S. suis*. The normal operation of various biological functions in bacteria depends on the biophysical integrity of the CM [38]. Thus, floxuridine may kill *S. suis* by disrupting the bacterial membrane.

More importantly, drug safety is an important indicator for clinical use. The cytotoxicity of floxuridine was evaluated using porcine kidney PK15 cells, and it was reported that floxuridine had a lower level of toxicity when compared with its potent antibacterial activity [28]. In addition, the long-term use of floxuridine can cause intestinal toxicity such as vomiting and diarrhea, but this toxicity can be diminished by changing the circadian rhythm of the infusion [39,40]. In this study, we used a single treatment to reduce side effects, and indeed, the mice experienced no side effects during the treatment. In addition, studies have shown that a single treatment is as effective as continuous treatment in mice [29]. Consistent with previous findings, floxuridine exhibited a strong antibacterial activity against *S. suis* in vitro and improved the survival rate in mice infected with *S. suis*. Thus, floxuridine was a potential antibacterial agent against *S. suis*.

Virulence-related genes play an important role in pathogenicity [41]. Therefore, inhibiting the activity of genes associated with virulence is one of the most effective ways to reduce the *S. suis* infections. Anti-virulence strategies have been proposed to prevent diseases caused by bacteria without killing bacterial growth [42]. In comparison with conventional antibiotics, the anti-virulence approach may be gentler on the development of resistance [43,44]. It has previously been reported that floxuridine has an inhibitory effect on the virulence of *Staphylococcus aureus* [29]. In this study, we found for the first time that floxuridine could significantly inhibit the hemolytic activity of an *S. suis* supernatant. We further revealed that floxuridine can bind to the SLY gene directly and inhibit the expression levels of virulence-related genes, *sly*, *fabps*, *gap* and *ef* included. As a critical virulence factor, *sly* is the only stimulus responsible for the activation and aggregation of platelets induced by *S. suis* [45]. Studies have shown that a high level of expression of SLY is associated with a high level of pathogenicity of the strain; moreover, the deletion of the *sly* gene can significantly reduce the mortality of mice [46]. *ef*, *fabps* and *gap* are adhesion-related genes [47,48], and the down-regulation of these genes indicates that floxuridine may inhibit the adhesion function of *S. suis*. Furthermore, a molecular docking assay was performed to verify the inhibitory effects of floxuridine on the SLY gene, and seven potential binding sites were demonstrated. Among the seven binding sites, some participated in the formation of hydrogen bonds or π-sigma with floxuridine. These results indicate that floxuridine exerts antivirulence potency by interacting with SLY’s active residues.

In conclusion, our results showed that the FDA-approved drug floxuridine exhibited potent antibacterial activities against *S. suis* both in vitro and in vivo. A mechanism study showed that floxuridine killed bacteria by disrupting the permeability of the CM. In addition, floxuridine can reduce the virulence of S. suis by reducing the expression of virulence genes. Therefore, floxuridine is a promising candidate for the development of novel antibiotic agents against *S. suis*.

## 4. Materials and Methods

### 4.1. Bacterial Strains and Drug

All bacteria strains used in this study are listed in Table 1. Nine clinical isolates of *S. suis* were obtained from pig lungs and were isolated and preserved by our laboratory. *S. suis* was cultured using tryptic soya broth (TSB) or plated on tryptic soya agar (TSA). Floxuridine (CAS: 50-91-9) was purchased form Yuanye Biotechnology (Shanghai, China).

### 4.2. Antimicrobial Activities Assay

The in vitro antimicrobial activities of floxuridine were evaluated via the microbroth dilution method, referring to the Clinical and Laboratory Standards Institute (CLSI) guidelines. Briefly, bacteria were inoculated in Mueller–Hinton broth (MHB) except for *S. suis*, which was cultured in MHB containing 5% fetal bovine serum. The drug was then serially diluted 2-fold in MHB and mixed with an equal volume of bacterial inoculum in a 96-well microtiter plate. Bacteria (5 × 10^5^ CFU/mL) were incubated with the drug at 37 °C for 18–20 h. The minimum inhibitory concentration (MIC) represents the minimum concentration that completely inhibits the growth of bacteria.

### 4.3. Time-Dependent Killing Assay

The overnight cultures of *S. suis* ATCC 43765 were diluted 1:100 in an MHB medium containing 5% fetal bovine serum and incubated at 37 °C with shaking at 180 rpm. *S. suis* ATCC 43765 was treated with 1 × MIC, 4 × MIC and 10 × MIC of floxuridine, respectively, and the time at which the drug was added was defined as 0 h. Samples were collected at 0, 1, 2, 4, 6, 8, 10 and 12 h. The bacterial suspension was serially diluted 10-fold and spread on LB agar plates. The colony counts were obtained after incubation at 37 °C for 16 h. 

### 4.4. Antihemolysin Activity Assessment

The effect of floxuridine on the hemolytic activity of *S. suis* was assessed. Briefly, resuspended *S. suis* were grown overnight with fresh TSB to an OD_600_ of approximately 0.5, and then various concentrations of floxuridine (0, 0.25, 1 and 25 μg/mL) were added for 6 h. The bacterial supernatant was collected via centrifugation, and sterile sheep red blood cells were added and incubated at 37 °C for 30 min. PBS (pH = 7.4) treated with or without Triton X-100 served as positive and negative controls, respectively. The hemolysis rate of floxuridine was assessed by measuring the absorption of hemoglobin at 543 nm. 

### 4.5. RT-PCR Analysis

Overnight, *S. suis* ATCC 43765 were diluted 10-fold in fresh TSB with 2.5 μg/mL of floxuridine. After incubation for 4 h at 37 °C, the total RNA was extracted using Trizol and then reverse-transcribed into cDNA. The 16S rRNA was used as the reference gene, and transcript levels of virulence-related genes (*ef*, *fabps*, *gapdh* and *sly*) were detected via a qPCR. The primers used in this study are listed in Table 2. The qPCR was performed using the SYBR PremeScript Mix (Takara, Beijing, China), and the PCR conditions were as follows: a two-step amplification method using 95 °C for 30 s, 40 cycles of 95 °C for 5 s and 58 °C for 40 s. The correlative expression levels of the virulence-related genes were calculated via the 2^−ΔΔCT^ method.

### 4.6. Molecular Docking Assay

The prediction of potential floxuridine and SLY binding sites was carried out via molecular docking. The structure of floxuridine was drawn using ChemDraw 16.0 software. The crystal structure of SLY was obtained from the Protein Data Bank (PDB entry: 3HVN) [51]. The docking procedure was carried out as previously described [52]. After the preparation of the ligand molecule and the protein, the LibDock docking mode was used, and the binding sites were shown using Discovery Studio 2019 Client. The binding energy was calculated using Autodock Vina 1.1.2 software.

### 4.7. Membrane Integrity Assay

Overnight cultures of *S. suis* ATCC 43765 cells were washed twice with PBS (pH = 7.4) and then resuspended in PBS (pH = 7.4) to an OD_600_ nm of 0.5. A final concentration of 10 nM of propidium iodide (PI) (Aladdin, China) was added to the resuspension and incubated for 20 min, followed by treatment with floxuridine (0.25, 1, 2.5 and 25 μg/mL). After 30 min of incubation, the fluorescence was measured at an excitation wavelength of 535 nm and an emission wavelength of 615 nm.

### 4.8. Membrane Potential Assay

The effect of floxuridine on the membrane potential (ΔΨm) of *S. suis* ATCC 43765 was assessed using the fluorescent probe DiSC_3_(5) (Thermo Scientific, Waltham, MA, USA). *S. suis* ATCC 43765 were washed with and resuspended in HEPES containing 20 mM of glucose and adjusted to an OD_600_ nm of 0.5. A final concentration of 5 μM DiSC_3_(5) was added to the cell suspension and incubated for 20 min, followed by the addition of floxuridine at various concentrations. Carbonyl cyanide 3-chlorophenylhydrazone (CCCP) was used as the negative control. After 30 min of incubation, the dissipated membrane potential of *S. suis* ATCC 43765 was measured at an excitation wavelength of 622 nm and an emission wavelength of 670 nm.

### 4.9. Scanning Electron Microscopy (SEM) Assay

*S. suis* ATCC 43765 cells were washed twice and resuspended in PBS (pH = 7.4). Various concentrations of floxuridine (0, 1 × MIC, 10 × MIC) were added to the cell suspension and incubated for 4 h. the cells were collected via centrifugation at 5000 rpm for 6 min and then resuspended in 2.5% glutaraldehyde overnight at 4 °C. The cell morphology was observed under a Hitachi Regulus 8100 SEM (Hitachi, Tokyo, Japan).

### 4.10. Mouse Infection Models

A mouse model of peritonitis was constructed in male BALB/C mice to evaluate the treatment effect of floxuridine. Briefly, 100 µL of *S. suis* SS3 containing 1.8 × 10^8^ CFU bacteria was injected intraperitoneally. After 1 h, the mice were treated with different concentrations of floxuridine. The number of mouse deaths was recorded daily over the next 5 d. The mice were euthanized via cervical dislocation after 5 d. The mouse organs were collected aseptically immediately after the death of the mouse. The mouse organs were collected aseptically, homogenized and plated to count bacterial numbers. Pathological changes in mice in different treatment groups were observed after the HE staining of different organs, including the liver, spleen, kidney, and lung.

### 4.11. Ethical Approval

Female BALB/C mice aged 6–8 weeks were purchased from the Guangdong Medical Lab Animal Center. The procedures for using animals were approved by the SCAU Animal Research Committees (2021b220). All animal studies were conducted in accordance with the SCAU Institutional Animal Welfare and Ethics Guidelines.

### 4.12. Statistical Analysis 

GraphPad Prism 8.0 was used for data analysis. All data were expressed as the means ± standard deviation (SD), and the *p*-values were evaluated via a one-way ANOVA among multiple comparisons or a t-test between two comparisons. * *p* < 0.05, ** *p* < 0.01.

## Figures and Tables

**Table 1:** The MIC detection results for different MLST types of *S. suis*.

Isolate	MLST	MIC (μg/mL)	MIC (μg/mL)
		Floxuridine	Tetracycline
ATCC 43765	-	0.25	1
SS3	7	0.5	64
SS30	25	0.12	64
SS14	27	0.25	1
SS37	242	0.12	64
SS12	850	0.06	64
SS25	308	0.5	2
SS26	839	0.12	8
SS35	28	0.12	64
SS40	87	0.25	16

**Table 2:** The primers used in this study.

Target	Primer	Sequence (5′-3′)	Source
16S rRNA	16S rRNA-F	CATCCATAACAGCCATACCAG	[49]
	16S rRNA-R	TAAACCACATGCTCCACCGC	
gapdh	gapdh-F	GCTGAAGAAGTAAACGCTGCT	[49]
	gapdh-R	GTCGCATCAAACAATGAACC	
ef	ef-F	TCCAATCACAGATCCAGATAGCG	[49]
	ef-R	CTGACCCATTTGGACCATCTAAG	
fbps	fbps-F	AACCATCTTGCCAGGCTCCAC	[50]
	fbps-R	CAGTTCAGAAGCCGTATCCCGAC	
sly	sly-F	TCATTCAGGTGCTTATGTTGCG	[50]
	sly-R	GAAGATTGCGAGCATTTCCTGG	

## Data Availability

The original data in this study are provided in the article.
